# 40 years of actigraphy in sleep medicine and current state of the art algorithms

**DOI:** 10.1038/s41746-023-00802-1

**Published:** 2023-03-24

**Authors:** Matthew R. Patterson, Adonay A. S. Nunes, Dawid Gerstel, Rakesh Pilkar, Tyler Guthrie, Ali Neishabouri, Christine C. Guo

**Affiliations:** ActiGraph LLC, 70 N Baylen St, Suite 400, Pensacola, FL USA

**Keywords:** Outcomes research, Diagnostic markers

## Abstract

For the last 40 years, actigraphy or wearable accelerometry has provided an objective, low-burden and ecologically valid approach to assess real-world sleep and circadian patterns, contributing valuable data to epidemiological and clinical insights on sleep and sleep disorders. The proper use of wearable technology in sleep research requires validated algorithms that can derive sleep outcomes from the sensor data. Since the publication of the first automated scoring algorithm by Webster in 1982, a variety of sleep algorithms have been developed and contributed to sleep research, including many recent ones that leverage machine learning and / or deep learning approaches. However, it remains unclear how these algorithms compare to each other on the same data set and if these modern data science approaches improve the analytical validity of sleep outcomes based on wrist-worn acceleration data. This work provides a systematic evaluation across 8 state-of-the-art sleep algorithms on a common sleep data set with polysomnography (PSG) as ground truth. Despite the inclusion of recently published complex algorithms, simple regression-based and heuristic algorithms demonstrated slightly superior performance in sleep-wake classification and sleep outcome estimation. The performance of complex machine learning and deep learning models seem to suffer from poor generalization. This independent and systematic analytical validation of sleep algorithms provides key evidence on the use of wearable digital health technologies for sleep research and care.

## Introduction

Sleep has intrigued researchers in both basic and clinical sciences for more than a century. With the advancement of sensor technology in the 1970s, sleep researchers started to experiment with the use of accelerometers worn on the wrist to objectively assess sleep and circadian patterns. This research grew rapidly in the 90s with improved sensor technology, increased storage size and improved usability of the devices. The rich body of sleep research using wearable devices has contributed substantially to our understanding on the importance of sleep to overall health^1^ with poor sleep linked to the progression of many diseases, including depression^2^, hypertension^3^, obesity^4^ and neurodegenerative diseases^5^. Since the 2010s, wearable devices have been increasingly used as digital heath technologies (DHT) to provide patient-centric clinical outcomes for drug development, where sleep outcomes represent one of the major use cases.

Sleep outcomes in clinical trials have traditionally been provided by polysomnography (PSG) and / or questionnaires. While PSG is the gold standard for assessing sleep physiology, its cost and burden can be prohibitive for large-scale deployment. The ecological validity of PSG-based sleep outcomes is also poor due to the need to attach a variety of sensors to the participant and the need for the participant to sleep in a supervised laboratory environment. Due to these limitations, some sleep studies use sleep diaries and questionnaires, but such sleep ratings are known to suffer from subjective and recall bias^6^. Wrist-worn accelerometer devices therefore offer an attractive solution for objective sleep assessments over multiple nights in the natural home environment. Such devices have low patient burden and can collect high-resolution data continuously for multiple weeks without recharging, thus minimizing the burden on the participants. To derive sleep outcomes, automated scoring algorithms are used to classify sleep and wake based on wrist acceleration. The first such algorithm was developed using simple linear regression and validated against PSG in 1982^7^, with the coefficients of the linear regression equation being updated in 1992^8^. The latter, referred to as Cole-Kripke, has become one of the most used sleep algorithms to date.

Due to technical limitations, early actigraphy devices would perform data reduction on the device to convert the raw acceleration data into activity counts and only save the latter^9^. Many legacy sleep algorithms such as Cole-Kripke thus use counts as input features for sleep-wake classification^8^. These legacy algorithms follow the same format of quantifying activity count-based features around the epoch of interest and applying them in a linear or logistic regression equation to make the binary prediction of sleep or wake^10,11^. More recently, with the availability of large sleep datasets, machine learning and deep learning methods were applied to sleep-wake classification. Using the Multi-Ethnic Study of Atherosclerosis (MESA) sleep dataset of 1817 participants^12^, deep learning models were shown to have higher sensitivity and specificity compared to legacy regression-based algorithms^13^.

With technological advancement and needs from the research community, manufacturers started to provide raw acceleration data from actigraphy devices, making it possible to develop sleep algorithms based directly on raw acceleration, not the aggregated activity counts. van Hees et al. (2015) used raw acceleration data to derive the angle of the forearm and developed a sleep-wake classification method based on the range of the angle over time^14^. This group later developed a random forest model on raw acceleration data and showed it to be more accurate than the original model and two legacy algorithms (Cole-Kripke and Sadeh)^15^.

To fully leverage the potential of wrist-worn wearables, the sleep research community can benefit from a systematic evaluation on the analytical validity of the various sleep algorithms developed and used for the last 40 years. Such evidence can support fit-for-purpose use of the proper algorithms in sleep research and therapeutics development. The current manuscript provides the first systematic comparison across simple regression to complex deep-learning models applied to either count or raw data. We assessed the performance of these algorithms in terms of classification accuracy and validity in estimating sleep outcomes such as WASO for direct relevance to clinical applications.

## Results

### Performance on Sleep-Wake Classification

**Fig. 1:**
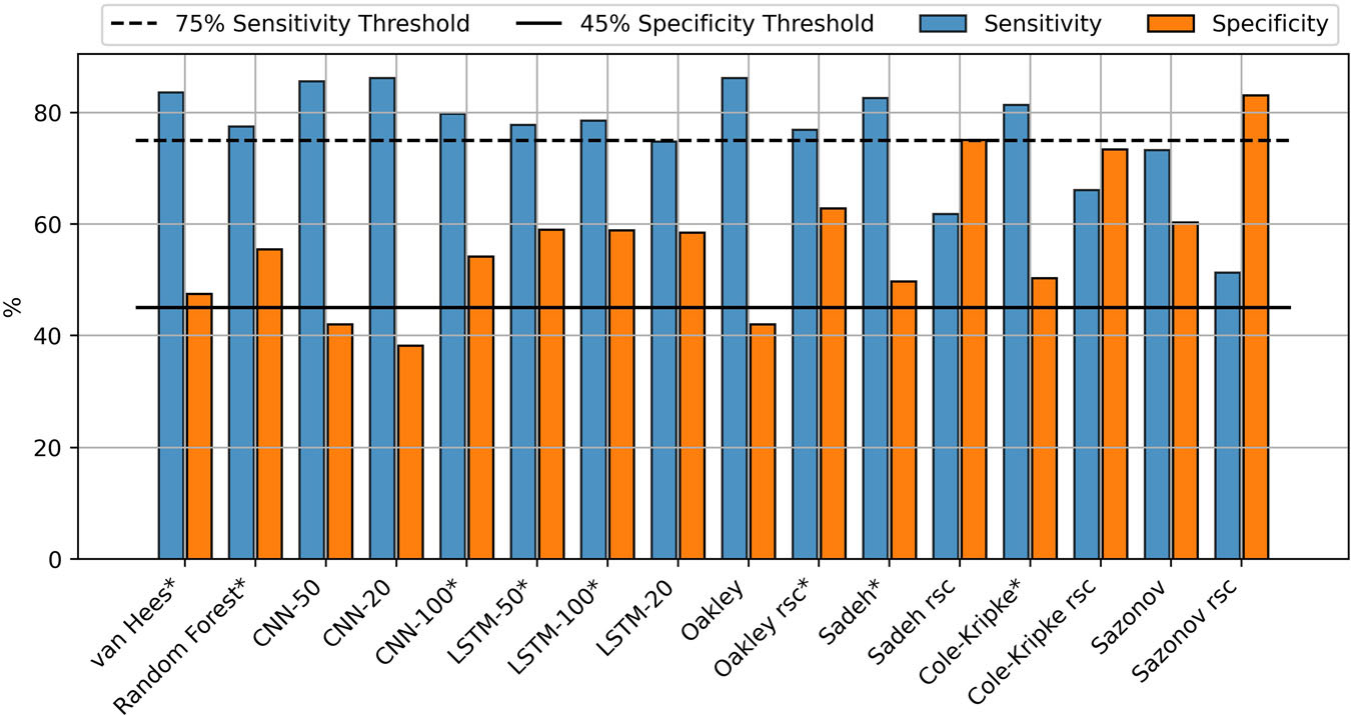
Sensitivity and specificity for each algorithm predicting sleep or wake compared to PSG. Sensitivity values are shown in blue and specific values are shown in orange. Algorithms that met the sensitivity and specificity thresholds are indicated with an asterisk.

Sleep-wake classification is an unbalanced classification problem, in that the datasets typically contain more sleep than wake. For such problems, the F1 score is typically used to rank algorithm performance, as it balances precision and sensitivity. However, the algorithms with the highest F1 scores had low specificity (CNN-50, Oakley, all Sleep) (Table 1). Since specificity, which represents classifying wake correctly, is essential to quantifying sleep disturbances, it should be given strong consideration in the evaluation. We thus selected only algorithms that passed minimal levels of sensitivity (75%) and specificity (45%) (Fig. 1; bold, Table 1). Among these algorithms, Oakley-rescore has the highest specificity at 62.8%, and van Hees has the best sensitivity of 83.6 and the highest F1 score of 79.1.

**Table 1:** Confusion matrix results for the binary classification problem of identifying sleep-wake from various algorithms compared to gold standard PSG annotations.

	Accuracy	Sensitivity	Specificity	Precision	F1
Reference					
All Sleep	69.2 (0.2)	100 (0.0)	0 (0.0)	69.2 (0.2)	79.6 (0.2)
All Wake	30.8 (0.2)	0 (0.0)	100 (0.0)	0 (0.0)	0 (0.0)
Raw Acceleration					
** van Hees**	**76.2 (0.1)**	**83.6 (0.2)**	**47.5 (0.2)**	**76.2 (0.2)**	**79.1 (0.2)**
** Random Forest**	**73.3 (0.1)**	**77.5 (0.2)**	**55.5 (0.2)**	**77.7 (0.2)**	**76.4 (0.2)**
Deep Learning Count					
CNN-50	77.3 (0.2)	85.6 (0.2)	42 (0.2)	76.1 (0.2)	79.6 (0.2)
CNN-20	76.2 (0.2)	86.2 (0.2)	38.2 (0.2)	74.9 (0.2)	79.4 (0.2)
** CNN-100**	**77 (0.2)**	**79.8 (0.2)**	**54.2 (0.2)**	**78.4 (0.2)**	**77.8 (0.2)**
** LSTM-50**	**76.3 (0.1)**	**77.8 (0.2)**	**59.0 (0.2)**	**78.8 (0.2)**	**77.1 (0.2)**
** LSTM-100**	**77.2 (0.1)**	**78.6 (0.2)**	**58.9 (0.2)**	**79.7 (0.2)**	**77.8 (0.2)**
LSTM-20	73.5 (0.1)	74.8 (0.2)	58.5 (0.2)	77.9 (0.2)	75.4 (0.2)
Legacy Count					
Oakley	75.1 (0.1)	86.2 (0.1)	42 (0.2)	75.1 (0.2)	79.2 (0.2)
** Oakley rsc**	**76.4 (0.1)**	**76.9 (0.2)**	**62.8 (0.2)**	**80.6 (0.2)**	**77.8 (0.2)**
** Sadeh**	**75.3 (0.1)**	**82.6 (0.2)**	**49.7 (0.2)**	**76.4 (0.2)**	**78.5 (0.2)**
Sadeh rsc	69.4 (0.1)	61.8 (0.2)	75.1 (0.2)	83 (0.2)	68.9 (0.2)
** Cole-Kripke**	**74.5 (0.1)**	**81.4 (0.2)**	**50.3 (0.2)**	**76.3 (0.2)**	**78 (0.2)**
Cole-Kripke rsc	71.8 (0.1)	66.1 (0.2)	73.4 (0.2)	82.5 (0.2)	72.2 (0.2)
Sazonov	71.3 (0.1)	73.3 (0.2)	60.3 (0.2)	78.2 (0.2)	74.8 (0.2)
Sazonov rsc	63.5 (0.1)	51.3 (0.2)	83.1 (0.2)	84.1 (0.2)	61.8 (0.2)

The mean values of all cases are presented with standard deviation in brackets. The top performing algorithms are shown in bold. *Rsc* rescore.

### Performance on sleep outcome estimation

**Fig. 2:**
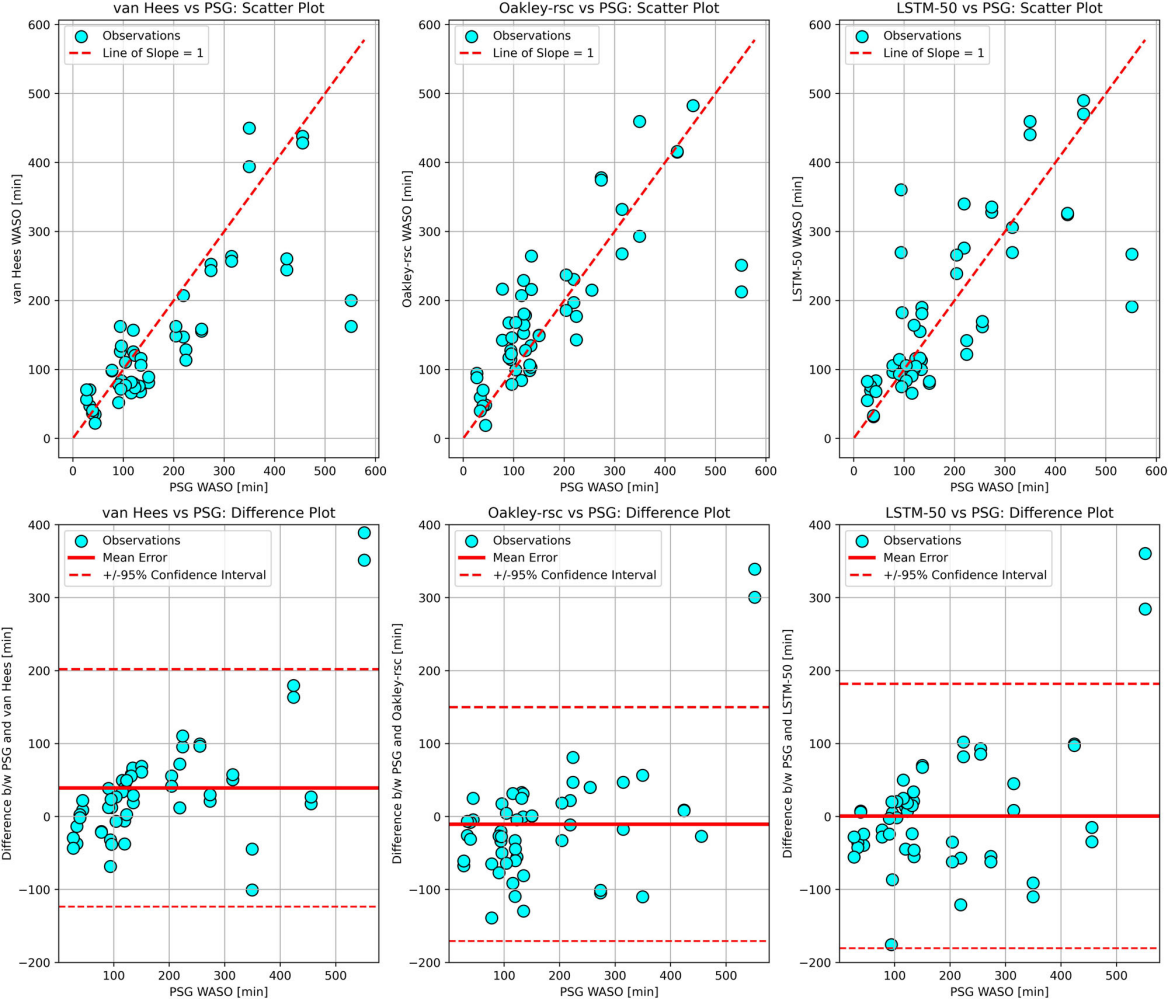
Bland-Altman validation plots for estimated WASO from the top performing algorithms in each category compared to PSG. The upper plots show the comparison of each algorithm estimated WASO to PSG WASO in circles, with a dashed line of slope equal to one representing where points would fall if the algorithm and PSG matched perfectly. The lower plots show the PSG WASO plotted against the difference between the algorithm and PSG for each data point. The solid horizontal line represents the mean error and the dashed horizontal lines represent the 95% confidence intervals of the differences.

For sleep studies, the goal of sleep-wake classification is to derive the sleep outcomes of interest. We thus examined the performance of the algorithms in estimating sleep outcomes using the Bland-Altman validation approach. Wake after sleep onset (WASO) is a commonly used sleep outcome. Among the selected algorithms, Oakley-rescore showed the lowest root mean squared error (RMSE) and narrowest confidence interval in estimating WASO as compared to the PSG-derived WASO (Table 2, Fig. 2). Cole-Kripke and van Hees also presented low RMSE scores and narrow confidence intervals. These three algorithms also showed much higher correlation with PSG-derived WASO than other algorithms. As for the more complex, machine learning models, the best performers amongst them (Random Forest, LSTM-50 and LSTM-100) showed low mean errors, but high RMSE scores, wide confidence intervals and low correlation with PSG-derived WASO. The performance of all the algorithms on the estimations of WASO, total sleep time (TST) and sleep efficiency (SE) showed similar patterns (Supplementary Tables 1 to 3).

**Table 2:** Bland-Altman statistics for wake after sleep onset (WASO) comparison between PSG and the sleep algorithms applied to wrist-based acceleration.

	ME	RMSE	Correlation	CI-95%+	CI-95%-	CI-width
Raw Acceleration						
van Hees	39.3	91.1	0.786	201.9	−123.4	325.3
Random Forest	1.0	93.0	0.717	184.9	−183.0	367.9
Deep Learning Count						
CNN-100	11.9	99.8	0.712	207.9	−184.1	392.0
LSTM-50	0.7	91.6	0.743	181.9	−180.5	362.4
LSTM-100	2.6	93.9	0.736	188.3	−183.1	371.4
Legacy Count						
Oakley rsc	−10.4	81.7	0.795	149.9	−170.7	320.6
Sadeh	33.6	93.6	0.756	206.3	−139.0	345.3
Cole-Kripke	28.4	90.4	0.770	198.1	−141.3	339.4

*ME* mean error, *RMSE* root mean squared error, *CI* confidence interval, *rsc* rescore.

## Discussion

Wrist-worn accelerometers are the most used wearable DHTs in clinical trials and research today. Accelerometer-based DHTs continue to play a central role in clinical research, thanks to the low cost, lightweight, long battery life and decades of research findings and datasets. The algorithms used to derive sleep outcomes from wrist acceleration data have been the focus of technical improvement and continue to evolve with the advancement of data science. Our systematic evaluation of the most common sleep algorithms developed over the past 40 years provides researchers with an evidence-based approach to use them effectively in sleep research.

Our findings suggest that the current application of machine learning and deep learning techniques to predict sleep-wake classification are not as robust in estimating sleep outcomes as simple heuristic (van Hees) and legacy regression models (Oakley-rescore and Cole-Kripke). This is surprising, as the deep learning and random forest models were trained on large datasets and thus would be expected to better model the complex relationship between wrist movements and sleep than simple models developed on smaller datasets^13,15^. The fact that these machine and deep learning models do not perform better may be due to the difference in activity counts used across sleep data sets, suboptimal model architectures, the intrinsic challenge with using motion data only to estimate sleep physiology and different PSG annotation styles between different data sets.

Computation of activity counts from raw accelerometer data is a common data reduction step from the early days of actigraphy. The conversion from raw acceleration to activity counts is not always well documented nor understood. Early studies presenting the legacy count algorithms did not provide any information about how the counts were obtained^7,8,10,16^. In addition, manufacturers might not disclose the way they derive counts from raw accelerometer data. The activity count calculation is a crucial step in the count-based sleep algorithms and differences in the counts would lead to differences in sleep-wake classifications. Despite this, the current research confirmed that legacy algorithms estimate sleep outcomes with high validity on counts computed using the open-source agcounts Python package^9^. The deep learning count-based algorithms were trained on proprietary counts from the MESA dataset and may not generalize to different types of counts as readily as the simple legacy count algorithms due to the model’s overfitting the PSG annotation style in the MESA dataset^13^. Due to the lack of available raw acceleration data in large data sets, no raw acceleration-based deep learning models have been presented in the literature.

The top-performing deep learning algorithms performed slightly worse than the much simpler heuristic and legacy algorithms. It is possible that the model architectures used in the current deep-learning algorithms may not be optimal^15^. In particular, the models are very simple, having only one layer of convolution filters or LSTM cells connected to a dense layer. Most deep learning models employ several layers (hence the term deep) which helps them capture more details from the training set. Their training also did not involve regularization techniques designed to avoid overfitting (and thus improve generalization) such as dropout or early stopping. The fact that the models were trained for only 30 epochs may limit their performance compared to heuristic algorithms^15^. In short, while deep learning algorithms hold promise, there is still work to be done.

Polysomnography (PSG) is considered the gold standard for sleep assessment and provides clinical diagnosis of sleep disorders such as apneas, hypopneas and rapid eye movement (REM) disorders^17^. Using PSG scoring as the ground truth for actigraphy sleep algorithms, however, has some intrinsic challenges. PSG measures physiological changes during sleep, while wrist actigraphy measures the movement of the distal forearm. Physiology and movement present highly structured and correlated patterns during sleep and wake cycles, which is the fundamental principle behind actigraphy use in sleep research. But the intrinsic difference between the two types of source signals means that there is a limit to how close one can be used to estimate the other. This does not mean wrist-based accelerometer assessment of sleep is inferior to PSG, as this method is superior in longitudinal and reliable assessment of sleep patterns in free-living environments. To facilitate the proper use of wrist accelerometer-based sleep outcomes, it may be necessary to interpret actigraphy-quantified sleep endpoints in their own right, and not expect them to perfectly match PSG.

Due to the subjective nature of PSG scoring, it may be difficult for a model to generalize between different data sets with different scorers. PSG needs to be scored by trained technicians to derive sleep outcomes. The scoring process takes 2–4 h to score one night of sleep and is also known to have high inter-rater variability, especially in pathological sleep populations^18^. To improve objectivity and reduce variability, the American Academy of Sleep Medicine (AASM) guidelines provide a series of rules that the PSG technician applies while they score raw PSG data^17^. For example, the scoring rule for wake is when more than 50% of the epoch contains an alpha rhythm (8–13hz) over the occipital region or eye blinks at 0.5 to 2hz or rapid eye movements associated with normal / high chin muscle tone or reading eye movements^17^. Such scoring criteria is inherently subjective and leaves room for different raters to score the same segment differently. Automated software packages have been developed to score PSG data; however, these are not considered gold-standard^18^. With the presence of high inter-rater variability, it may be difficult for a model trained on the relationship between PSG scoring and movement patterns to generalize robustly to different data sets scored by different raters. Deep learning and machine learning models run the risk of overfitting to data set-specific PSG annotator style if they do not include proper model architecture to enhance generalization.

While this work is the first systematic comparison across simple regression to complex acceleration-based machine learning sleep algorithms, a subset of these models has been evaluated in previous literature. Sundararajan et al. 2021 reported slightly worse results for Sadeh (F1 68.1 to 78.5%), Cole-Kripke (F1 67.5 to 78.0%), van Hees (F1 70.1 to 79.1%) and Random Forest (F1 73.9 to 76.4%) than the present study^15^. There are several potential reasons for this difference. One, no data was dropped in the current study. Sundararajan et al. 2021 had 24 participants in their test set, while the current work used all 28 participants from the Newcastle PSG dataset. Second, accuracy, sensitivity and other statistics were calculated for each subject and then averaged in the current work, whereas Sundararajan et al. 2021 combined all epochs from all subjects together and calculated the evaluation metrics. The advantage of averaging evaluation metrics from each participant is that a Bland-Altman style validation analysis can be performed on the sleep outcomes.

Rescoring is a series of heuristic rules that was developed in conjunction with the original legacy sleep algorithm to rescore periods as wake or sleep based on the length of a period and the length of the surrounding periods^7^. Previous research showed that rescoring improved performance for all legacy algorithms on the MESA data set^13^. However, on the Newcastle PSG data set, rescoring resulted in poorer performance for Cole-Kripke (RMSE 13.0 to 18.5), Sadeh (RMSE 13.6 to 23.4) and Sazonov (RMSE 14.5 to 30.5) algorithms, while it improved performance for the Oakley algorithm (RMSE 15.9 to 12.7). In both studies rescoring decreased sensitivity and increased specificity, so algorithms with high sensitivity and low specificity to begin with were improved with rescoring and algorithms with reasonable sensitivity and specificity to begin with were made worse with rescoring.

Supplementary Table 1 summarizes sensitivity, specificity and accuracy from previous studies presenting algorithms to predict sleep-wake from wrist accelerometry. The Sadeh model on the Sadeh data set had the highest specificity and accuracy of all other model / data set combinations, however the Sadeh data set consisted of only healthy sleepers. On the MESA data set, rescoring improved performance for the Sadeh, Cole-Kripke and Oakley algorithms. The van Hees and Sundararajan (random forest) algorithms could not be run on MESA because they require raw acceleration and the MESA data set contains only activity counts.

The current work has several limitations. Since the PSG scoring process was not detailed in the open-source Newcastle PSG dataset, we do not know how it was performed. The test data set in the current work was from one PSG study, future work should consider using multiple PSG studies as test data sets, to ensure generalizability to different PSG annotation styles. The challenge with this currently is that many open-source PSG data sets (MESA, STAGES) only include activity count data and not raw acceleration, making it impossible to test the raw acceleration-based algorithms. The current work considered algorithms that used acceleration only. Heart rate and other physiological signals have the potential to improve sleep classification and staging, however, the trade off with more sensors is a decrease in battery life^19^. This is an important area for future research.

## Methods

### Sleep dataset

PSG data was obtained from the open-source Newcastle PSG dataset^14^. The study design included ethics approval from the NRES North East Sunderland ethics committee (12/NE/0406) and participants provided written informed consent. PSG data was collected from 28 adult patients (11 female). Mean age of the participants was 44.9 years (14.9 years standard deviation). Concurrent wrist accelerometry data (GENEActiv, Kimboloton, UK) was collected at 85.7hz. All 28 patients had complete data from a left wrist accelerometer and 27 patients had complete data from a right wrist accelerometer. A single night PSG (Embletta, Denver) was performed using a standard procedure that included electroencephalogram (leads C4-A1 and C3-A2), video recording, bilateral eye movements, oxygen saturation, bilateral anterior tibialis EMG, abdominal and chest inductance bands, and submental EMG. All sleep stages were scored according to standard AASM criteria^20^. Twenty of the participants had at least one sleep disorder. Sleep disorders included idiopathic hypersomnia, restless leg syndrome, sleep apnea, narcolepsy, sleep paralysis, nocturia, obstructive sleep apnea, REM sleep disorder, parasomnia and insomnia.

### Accelerometer data processing

Raw tri-axial acceleration data was calibrated using GGIR, an R-package to process multi-day raw accelerometer data^21^. Analysis was performed by creating scripts in Python (v 3.8.13) that called each algorithm. For each algorithm, sleep-wake classifications were found in 30-second, non-overlapping windows and compared to the PSG-annotated sleep stages. All algorithms are summarized in Table 1. In addition, the random forest model presented in Sundararajan et al. 2021 was included. For the van Hees et al. 2015 algorithm, a python implementation of the algorithm described in the paper was used^14^. Table 3.

**Table 3:** Summary of the compared algorithms.

Paper	Model Type	Input	Model Complexity	Description
Van Hees	Heuristic	Acceleration	Low	Considers range of angle-z over 5 min window to classify it as sleep or wake
Sundararajan	Random Forest	Acceleration	High	Random forest machine learning model trained on 136 sleep patients from two different data sets
Palotti	CNN	Counts	High	Deep neural network trained on MESA data set (training *n* = 1454) using a convolutional neural network layer
Palotti	LSTM	Counts	High	Deep neural network trained on MESA data set (training *n* = 1454) using a long-short term memory layer
Cole-Kripke	Regression	Counts	Medium	Commonly used linear regression model
Oakley	Regression	Counts	Medium	Linear regression model with a trained threshold
Sadeh	Regression	Counts	Medium	Commonly used linear regression model
Sazonov	Regression	Counts	Medium	Logistic regression model trained on infants from sensor on diaper location

For the count-based algorithms, activity counts were calculated by first downsampling the raw acceleration data to 40hz by taking the mean in 25 milli-second windows and then using the open-source Python agcounts package to obtain activity counts in 30 s epochs^9^. The legacy count-based algorithms implemented were Cole-Kripke^8^, Oakley^10^, Sadeh^16^ and Sazonov^11^. In addition, Long Short-Term Memory (LSTM) and convolutional neural network (CNN) with sequences of 20, 50, and 100 samples were implemented with the same weights provided in Palotti et al.^13^. For the deep learning algorithms, the activity counts were combined by taking the 6^th^ root of the sum of the squared 3 axes values, then the values were normalized. Directly using the scaler from the MESA training set resulted in poor performance, likely due to differences in activity counts.

A rescoring was implemented on the legacy count-based algorithms that was originally proposed in Webster et al. 1982 and was implemented more recently in Palotti et al.^7,13^. The rescoring was only applied to the legacy algorithms as it was developed for the legacy algorithms and Palotti et al. 2019 showed that rescoring did not improve deep learning models performance. Original performance statistics from all algorithms are reported in Supplementary Table 4.

### Statistics

A confusion matrix was obtained for predictions from each algorithm for each subject compared to the gold standard PSG sleep stages at 30-second epochs. An epoch was true positive (TP) if both the PSG and the algorithm prediction labelled it sleep, an epoch was true negative (TN) if both the PSG and algorithm prediction were wake, an epoch was false positive (FP) if the PSG was wake and the algorithm prediction was sleep, finally, an epoch was false negative (FN) if the PSG was sleep and the algorithm prediction was wake. These equations were used to calculate the following statistics:

1 $$Sensitivity = \frac{{True\,Positives}}{{True\,Positives + False\,Negatives}}$$

2 $$Specificity = \frac{{True\,Negatives}}{{True\,Negatives + False\,Positives}}$$

3 $$Precision = \frac{{True\,Positives}}{{True\,Positives + False\,Positives}}$$

4 $$F1\,Score = 2*\frac{{Precision\,*\,Sensitivity}}{{Precision + Sensitivity}}$$

sensitivity, Eq. (1), was calculated as the percentage of PSG scored sleep that the algorithm scored correctly. Specificity, Eq. (2), was calculated as the percentage of PSG scored wake, that the algorithm scored correctly. Precision, Eq. (3), was calculated as the percentage of algorithm detected sleep that was correct according to PSG. F1 score, Eq. (4), was calculated as the harmonic mean of sensitivity and precision. Wake after sleep onset (WASO) was calculated by summing the amount of wake within the sleep period. Sleep efficiency (SE) was calculated as the percentage of sleep within the period in which there were PSG annotations. Total sleep time (TST) was calculated as the sum of sleep epoch classifications per night.

A Bland-Altman style validation statistical approach was applied to the sleep endpoints WASO, TST and SE because this provides validation statistics that are specific to the sleep endpoints that are commonly used in clinical research^22^. Mean error (ME) was calculated as the average of the differences between the predicted measure and the PSG derived measure for all patients. Mean error indicates if there is a bias in a model output. Root mean squared error (RMSE) was calculated as the square root of the squared mean error and is useful because positive and negative values cannot cancel each other out. Pearson’s correlation coefficient is calculated as well as the 95% confidence intervals, which is the range for which 95% of the differences between the predicted endpoint and the PSG scored endpoint exist^22^.

### Reporting summary

Further information on research design is available in the Nature Research Reporting Summary linked to this article.

## Supplementary information

Supplementary material

REPORTING SUMMARY

## Data Availability

The Newcastle Polysomnography dataset can be found at https://zenodo.org/record/1160410#.YynNvOzMJz8.

## Code availability

The Python based analysis code for this paper can be found at https://github.com/actigraph/Sleep-Wake-Classification.
