# Supplementary material for: 40 years of actigraphy in sleep medicine and current state of the art algorithms
Source: NPJ Digit Med. 2023 Mar 24;6:51. doi: 10.1038/s41746-023-00802-1 (PMC10039037; doi:10.1038/s41746-023-00802-1)
Supplement: Supplementary file 1 — Supplementary material [file 41746_2023_802_MOESM1_ESM.pdf]

Supplementary Material

	ME	RMSE	Correlation	CI-95%+	CI-95%-	CI-width
**All Sleep or Wake**	min	min		min	min	min
All Sleep	180.1	223.9	na	443.1	-83.0	526.1
All Wake	-391.3	409.4	0.490	-153.1	-629.6	476.5
**Raw Acceleration Algorithms**						
van Hees	39.3	91.1	0.786	201.9	-123.4	325.3
Random Forest	1.0	93.0	0.717	184.9	-183.0	367.9
**Deep Learning Count Algorithms**						
CNN-50	52.7	110.1	0.713	243.8	-138.4	382.2
CNN-20	61.6	111.7	0.723	245.8	-122.7	368.5
CNN-100	11.9	99.8	0.712	207.9	-184.1	392.0
LSTM-50	0.7	91.6	0.743	181.9	-180.5	362.4
LSTM-100	2.6	93.9	0.736	188.3	-183.1	371.4
LSTM-20	-10.6	86.4	0.766	159.1	-180.3	339.4
**Legacy Count Algorithms**						
Oakley	59.8	107.7	0.748	236.9	-117.3	354.2
Oakley rsc	-10.4	81.7	0.795	149.9	-170.7	320.6
Sadeh	33.6	93.6	0.756	206.3	-139.0	345.3
Sadeh rsc	-88.3	133.2	0.693	108.9	-285.6	394.5
Cole-Kripke	28.4	90.4	0.770	198.1	-141.3	339.4
Cole-Kripke rsc	-66.9	105.9	0.788	95.4	-229.3	324.7
Sazonov	-21.3	90.3	0.755	152.3	-194.9	347.2
Sazonov rsc	-143.7	169.3	0.739	33.5	-320.8	354.4

Supplementary Table 1 – Bland-Altman statistics for wake after sleep onset comparison between PSG and the various algorithms to predict it from wrist-based acceleration. ME – mean error, RMSE – root mean squared error CI – confidence interval and rsc – rescore.

	ME	RMSE	Correlation	CI-95%+	CI-95%-	CI-width
**All Sleep or Wake**	%	%		%	%	%
All Sleep	-30.8	37.2	na	10.8	-72.3	83.1
All Wake	69.2	72.4	na	110.8	27.7	83.1
**Raw Acceleration Algorithms**						
van Hees	-5.7	13.0	0.839	17.4	-28.8	46.2
Random Forest	0.9	15.2	0.717	30.8	-29.0	59.8
**Deep Learning Activity Count Algorithms**						
CNN-50	-7.8	16.9	0.784	21.7	-37.4	59.2
CNN-20	-9.5	16.7	0.797	17.6	-36.6	54.2
CNN-100	-0.6	16.4	0.764	31.9	-33.0	64.9
LSTM-50	1.3	15.1	0.779	31.0	-28.4	59.5
LSTM-100	1.0	15.5	0.779	31.7	-29.6	61.3
LSTM-20	3.2	14.0	0.791	30.3	-23.8	54.1
**Legacy Activity Count Algorithms**						
Oakley	-9.4	15.9	0.791	16.0	-34.8	50.8
Oakley rsc	2.8	12.7	0.827	27.3	-21.6	48.9
Sadeh	-4.8	13.6	0.798	20.3	-29.9	50.3
Sadeh rsc	16.7	23.4	0.708	49.0	-15.5	64.5
Cole-Kripke	-3.9	13.0	0.806	20.7	-28.5	49.2
Cole-Kripke rsc	12.9	18.5	0.795	39.0	-13.2	52.1
Sazonov	4.8	14.5	0.762	31.9	-22.2	54.1
Sazonov rsc	26.3	30.5	0.689	57.1	-4.6	61.6

Supplementary Table 2 – Bland-Altman statistics for sleep efficiency comparison between PSG and the various algorithms to predict it from wrist-based acceleration. ME – mean error, RMSE – root mean squared error CI – confidence interval and rsc – rescore.

	ME	RMSE	Correlation	CI-95%+	CI-95%-	CI-width
**All Sleep or Wake**	min	min		min	min	min
All Sleep	-180.1	223.9	0.271	83.0	-443.1	526.1
All Wake	391.3	409.4		629.6	153.1	476.5
**Raw Acceleration Algorithms**						
van Hees	-39.3	91.1	0.792	123.4	-201.9	325.3
Random Forest	-1.0	93.0	0.719	183.0	-184.9	367.9
**Deep Learning Activity Count Algorithms**						
CNN-50	-52.7	110.1	0.777	138.4	-243.8	382.2
CNN-20	-61.6	111.7	0.768	122.7	-245.8	368.5
CNN-100	-11.9	99.8	0.777	184.1	-207.9	392.0
LSTM-50	-0.7	91.6	0.788	180.5	-181.9	362.4
LSTM-100	-2.6	93.9	0.791	183.1	-188.3	371.4
LSTM-20	10.6	86.4	0.787	180.3	-159.1	339.4
**Legacy Activity Count Algorithms**						
Oakley	-59.8	107.7	0.721	117.3	-236.9	354.2
Oakley rsc	10.4	81.7	0.796	170.7	-149.9	320.6
Sadeh	-33.6	93.6	0.746	139.0	-206.3	345.3
Sadeh rsc	88.3	133.2	0.699	285.6	-108.9	394.5
Cole-Kripke	-28.4	90.4	0.746	141.3	-198.1	339.4
Cole-Kripke rsc	66.9	105.9	0.778	229.3	-95.4	324.7
Sazonov	21.3	90.3	0.724	194.9	-152.3	347.2
Sazonov rsc	143.7	169.3	0.693	320.8	-33.5	354.4

Supplementary Table 3 – Bland-Altman statistics for total sleep time (TST) comparison between PSG and the various algorithms to predict it from wrist-based acceleration. ME – mean error, RMSE – root mean squared error CI – confidence interval and rsc – rescore.

Paper	Model	Dataset	n	Sensitivity	Specificity	Accuracy
[16]	Sadeh	Sadeh	26	96.0	75.2	91.9
[8]	Cole-Kripke	Cole-Kripke	21	96.1	60.0	87.9
[8]	Cole-Kripke rescore	Cole-Kripke	21	95.2	64.5	88.3
[14]	van Hees	Newcastle	28	91	45	83
[15]	Sundararajan	Newcastle	24	89.7	58.9	nr
[13]	Sadeh	MESA	363	98.3	38.3	73.4
[13]	Sadeh rescore	MESA	363	92.8	59.4	79.1
[13]	Cole-Kripke	MESA	363	96.7	45.0	75.4
[13]	Cole-Kripke rescore	MESA	363	89.9	65.7	80.2
[13]	Oakley	MESA	363	96.9	41.2	73.9
[13]	Oakley rescore	MESA	363	92.8	59.8	79.3
[13]	LSTM-100	MESA	363	91.4	69.9	83.1
[13]	CNN-100	MESA	363	91.7	68.8	82.9
[13]	LSTM-50	MESA	363	90.5	70.1	82.7
[13]	CNN-50	MESA	363	92.0	67.6	82.5
[13]	CNN-20	MESA	363	90.9	66.5	81.4
[13]	LSTM-20	MESA	363	92.0	65.0	81.3

Supplementary Table 4 – Summary of validation statistics from the previous studies presenting algorithms to predict sleep-wake from wrist acceleration. Nr – not reported.
